# Editorial Chit-Chat

**Published:** 1875-01

**Authors:** 


### Editorial Chit-Chat.

FIVE CENTS EXTRA—this **time, for** postage.

SNOW.—O, for "snow, snow, the beautiful snow."

THE ENVELOPE.—It will be found in the present number, in which to enclose your subscription for the new year. Do not forget to enclose 5 cents extra, with which to prepay the postage.

A WONDERFUL LAKE.—Mr. Beecher, in one of his Friday evening lectures, mentioned a lake in Sullivan county, Pa., possessed of wonderful acoustic qualities, where he could paddle a boat to the middle of the lake—a half mile or more from shore—and plainly hear every word of ordinary toned conversation upon the shore. Even the dip of his paddle and the ripple of the water could be distinguished by those on shore, a half mile away.

DOCTOR LUKE.—We have been in attendance upon the excellent and instructive lectures of Mr. Beecher's upon the "Authors of the New Testament," and were surprised to learn that Luke was a "traveling doctor." We are sorry for this, because we had formed a good opinion of Luke and his writings, and dislike to think of him as a traveling charlatan. Then, to think that Dr. Luke should violate the "code," rendering himself ineligible to membership in the Chemung County Medical Society, is too bad for anything.

THE CENTENNIAL.—Philadelphia is making great preparation for the celebration of the Centennial. That the affair will be a magnificent success cannot be doubted when we come to witness the enthusiasm with which her citizens enter into the project, and the magnificent and immensely capacious buildings they are erecting for the purposes of the exhibition. A visit to Philadelphia, even at this early day, will convince the stranger that the old Quaker city people intend having a "big time."

ONLY A FLOWER.—On the 15th of November, while strolling over our hills in search of "ground pine," with our botanical friend from Canandaigua, we found a beautiful little white flower in full bloom. It looked so innocent and lonely there, among the cold bleak rocks, that we plucked it, intending to transfer it to the warmer temperature of our parlor. Presently we encountered our friend, who, the moment he discovered our exquisite boquet, exclaimed "an anemone! an anemone! and in bloom in November! Never heard of such an occurrence before! Why, that flower blooms early in the spring. "Where did you find it? Let us look for more." These, with other exclamations of admiration and astonishment, were the salutations which greeted our wild flower boquet. As the finding of the anemone, in bloom, during November, seems to be an unheard of occurrence in this latitude, we have made a note of it, in accordance with the wishes of our friend before mentioned.

PLATE GLASS.—We have a new door—in it is a large plate glass—it was not there aforetime, but contained common sheet glass—half the time, when not broken by the slam of the door. Well, so frequently was this glass broken that, the old cat made it a custom to jump through the door and soon taught every one of her eleven kittens to do the same. The children would cry out their salutaions through this opening, poking their saucy little heads through the place where the glass should be. But they came to grief, both they, the old cat and her kittens. You see, our children and kittens were not brought up behind the luxury of plate glass, therefore were excusable for not comprehending the clearness of that article; hence, when our new door with its beautiful plate glass was in position, it became startling, and somewhat amusing, to see the old cat leap for that apparent aperture, only to find herself flat on her back, contemplating her surprise from the wrong side of the door, and getting up, squinting at the supposed opening, and calculating the distance from the floor, she tried it again, this time resulting in a bloody nose and a worse repulse than before, after which she quietly walked away and contemplated the phenomena from our neighbor's fence. We thought we detected a quiet smile of satisfaction upon her countenance as her several kittens attempted the same freak with varying results. This was all very well for the cats and kittens, but when our children and their playmates appeared before us with bumped noses and bruised heads, the matter became serious, so that now we display a card upon the clear surface of this beautiful glass, bearing this inscription:—"Beware of Your Nose;" since which time cat, kittens and babies have ceased their gymnastics in that locality.

